# Allergic sensitization to lipocalins reflects asthma morbidity in dog dander sensitized children

**DOI:** 10.1002/clt2.12149

**Published:** 2022-05-02

**Authors:** Ulrika Käck, Marianne van Hage, Hans Grönlund, Gunnar Lilja, Anna Asarnoj, Jon R. Konradsen

**Affiliations:** ^1^ Department of Clinical Science and Education Södersjukhuset Karolinska Institutet Stockholm Sweden; ^2^ Sachs' Children and Youth Hospital Södersjukhuset Stockholm Sweden; ^3^ Department of Medicine Solna Division of Immunology and Allergy Karolinska Institutet and Karolinska University Hospital Stockholm Sweden; ^4^ Department of Clinical Neuroscience Karolinska Institutet Stockholm Sweden; ^5^ Astrid Lindgren Children's Hospital Karolinska University Hospital Stockholm Sweden; ^6^ Department of Women's and Children's Health Karolinska Institutet Stockholm Sweden

**Keywords:** allergen molecules, asthma, can f, children, dog allergy, lipocalins, nasal provocation testing

## Abstract

**Background:**

Sensitization to dog is an important risk factor for asthma in children, but the clinical relevance of IgE to available dog‐ and furry animal allergen molecules is uncertain.

**Methods:**

Spirometry, methacholine challenge, fraction of exhaled nitric oxide, nasal challenge with dog extract and questionnaires were performed in 59 dog‐sensitized children (age 10–18 years). Serum IgE to dog‐, cat‐, horse extracts and the allergen molecules Can f 1–6, Fel d 1, Fel d 2, Fel d 4 and Equ c 1 were evaluated.

**Results:**

Median numbers of positive IgE results to furry animal allergen molecules among children without asthma was 3, with asthma 5.5 and with troublesome asthma 9 (asthma vs. no asthma; *p* = 0.039; troublesome asthma vs. no asthma; *p* = 0.009). The odds ratio for asthma if sensitized to any lipocalin was 7.2 (95% confidence Interval: 1.44–35.9). Children with troublesome asthma had higher IgE levels to the lipocalins Can f 2, Can f 4 and Can f 6 compared to the rest of the study population (44 vs. 4.1 kU_A_/L, *p* = 0.015; 5.8 vs. 0.9 kU_A_/L, *p* = 0.018 and 1.3 vs. 0.7 kU_A_/L, *p* = 0.03 respectively). Furthermore, a positive nasal challenge was more common among children with troublesome asthma (83% vs. 36%, *p* = 0.036).

**Conclusions:**

Polysensitization to furry animal allergens and lipocalins is associated with asthma in dog‐sensitized children. Children with troublesome asthma have higher IgE levels to several dog lipocalins than other dog sensitized children.

**Key message:**

Polysensitization to furry animal allergens and high IgE levels to the dog lipocalins Can f 2, Can f 4 and Can f 6 is associated with asthma severity in dog dander sensitized children. Molecular allergy diagnostics may thus help the clinicians to evaluate the impact of allergic sensitization on asthma morbidity.

## 1 INTRODUCTION

Allergic sensitization to dog dander, which has increased in recent decades, is a well‐established risk factor for asthma in children.^1^, ^2^ However, the detection of serum IgE antibodies (IgE) to dog dander has limitations as the rate of asymptomatic sensitization is high,^3^ and the content of allergens in dog dander extracts varies.^4^, ^5^

Analysis of IgE to the dog allergen molecules Can f 1, Can f 2, Can f 4 and Can f 6, belonging to the lipocalin family, to Can f 3, the dog serum albumin, and to Can f 5, the prostatic kallikrein, offers improved opportunities to shed light on associations between asthma and allergic sensitization at the molecular allergen level.^6^ Molecular allergology has been suggested to be particularly useful in patients with polysensitization^7^, ^8^ and/or severe asthma.^9^

Population based studies have demonstrated that polysensitization and high‐titer sensitization to furry animal allergens are associated with persistent asthma.^1^, ^10^ Moreover, we have previously shown that polysensitization to the furry animal allergen molecules lipocalin, kallikrein and secretoglobin is associated with increased bronchial inflammation in children with severe asthma.^11^

We have previously investigated associations between a nasal challenge with dog dander extract and sensitization to dog allergen molecules among dog dander sensitized children and found that polysensitization is associated with a positive nasal challenge.^12^ The primary objective of this study was to investigate the relative importance of IgE reactivity to all six clinically available dog allergen molecules in relation to asthma among dog dander sensitized children. As poly‐sensitization to furry animal allergen molecules has shown to be common in children we also included clinically available cat and horse allergen molecules.^10^ To our knowledge, no previous study has included analysis of all known dog lipocalins in relation to asthma. In addition, we investigate sensitization in relation to the severity of asthma, evaluated by asthma control, markers of airway inflammation and bronchial hyperresponsiveness.

## 2 METHODS

### 2.1 Patient population

Sixty patients aged 10–18 years with confirmed sensitization to dog dander (skin prick test wheal size > 3 mm or serum IgE antibodies to dog dander ≥0.10 kU_A_/L) were included from pediatric outpatient clinics in the Stockholm area, regardless of history of clinical symptoms to dog. One patient did not complete the examinations and was excluded. The study protocol was approved by the Regional Ethics Committee of Karolinska Institutet, Stockholm (Dnr 2014/1453‐31/4). Written informed consent was obtained from participants and their parents.

All children and their parents were interviewed regarding clinical history, current symptoms, symptom triggers (exposure to dog, cat and horse, pollen and non‐allergic triggers such as infections and exercise) and medications for asthma and rhinitis according to a modified version of the standardized questionnaire employed in the Environmental and Childhood Asthma Study.^13^ Asthma control was assessed according to the Pediatric Asthma Control Test (ACT) among children 10–11 years of age (maximum score 27) and ACT for individuals above the age of 12 (maximum score 25). A score below 20 indicates deficient asthma control for both tests.^14^, ^15^

*Asthma* was defined as reported physician's diagnosis of asthma verified by review of medical charts. *Troublesome asthma* was defined as asthma diagnosis in combination with exhaled fraction of exhaled nitric oxide (FeNO) > 35 ppb, Methacholine PD20 < 2 μmol and ACT score < 20.

### 2.2 Exhaled nitric oxide, pulmonary function and assessment of bronchial hyper responsiveness

A NIOXTM analyzer (Aerocrine AB) was used to measure the FeNO, in accordance with international guidelines.^16^ A FeNO level above 20 ppb was considered elevated and above 35 ppb was considered high.^17^ Spirometry was performed using a Vitalograph^®^ 2120 (Vitalograph^®^, Ennis, Ireland), using the reference values reported by Polgar.^18^ Bronchial hyperresponsiveness to methacholine was assessed utilizing a Spira nebulizer (Spira Respiratory Care Center, Hämeenlinna, Finland). The dose of inhaled methacholine leading to a 20% drop in FEV1 (PD20) was calculated.^19^

### 2.3 Nasal provocation testing

Nasal provocation testing (NPT) was performed as previously described^12^ with a commercially available dog dander extract, Aquagen 100,000 SQ‐U/ml (ALK‐Abello, Copenhagen, Denmark) according to a modified Lebel protocol.^20^

### 2.4 Blood analysis

Blood samples were taken and the white blood cell count was performed. IgE antibodies against dog‐ cat‐ and horse dander extract, and the dog allergen molecules Can f 1, Can f 2, Can f 3, and Can f 5 were analyzed by ImmunoCAP. Can f 4 and Can f 6 were produced as recombinant proteins and analyzed by Streptavidin ImmunoCAP.^21^, ^22^

Sera that scored positive (IgE ≥ 0.10 kU_A_/l) for cat or horse extracts were further analyzed for IgE against allergen molecules from cat (Fel d 1, Fel d 2, Fel d 4) and horse (Equ c 1).

All IgE determinations were analyzed by using the ImmunoCAP System (Phadia AB/Thermo Fisher Scientific) according to the manufacturer's instructions. Results are presented as kilounits of allergen per liter, where the cutoff for allergen specific IgE was ≥0.10 kU_A_/L.

*Polysensitization* refers to the presence of specific IgE to more than one furry animal allergen molecule.

### 2.5 Statistics

Categorical data were compared by using the Chi 2 test or Fisher exact test, where appropriate. IgE levels were log‐transformed to obtain normal distribution. *t*‐test was used for group comparisons of normally distributed continuous variables and Mann‐Whitney for skewed continuous variables and for comparisons of the degree of polysensitization (ordinal variables) between children with no asthma, asthma and troublesome asthma. *P*‐values below 0.05 were considered significant. Bonferroni correction for multiple testing with single allergens was performed. Odds Ratios for having asthma compared to rhinitis only in relation to sensitization were estimated using logistic regression and 95% confidence intervals (CI). All statistical analyses were performed with Stata statistical software (release 14.2; StataCorp).

## 3 RESULTS

### 3.1 Clinical characteristics

Fifty of 59 dog dander sensitized children had asthma. Among the 50 children with asthma, 30 reported asthma triggered by dog exposure of whom six had troublesome asthma. The majority of children with asthma also reported allergic rhinitis (48/50; 96%), of whom 70% (*n* = 35) reported rhinitis triggered by dog exposure. Nine of the dog dander sensitized children did not have asthma, but allergic rhinitis only, of whom 5 reported rhinitis triggered by dog. Children with asthma showed a trend towards reacting more frequently upon NPT with dog dander extract compared to the non‐asthmatic children (46% vs. 11%, *p* = 0.07). Furthermore, asthma diagnosis was associated with a reduced score on the ACT and pronounced bronchial hyperreactivity (Table 1). There were no significant differences in exposure to dogs or cats at home between the groups.

**TABLE 1:** Clinical characteristics of included dog dander sensitized children with and without asthma

Variable	Dog sensitized: Asthma (*n* = 50)	Dog sensitized: No asthma (*n* = 9)	*p*‐Value
Age, mean (SD)	13.1 (2.29)	13.2 (2.77)	0.85
Gender, male *n* (%)	31 (62)	7 (78)	0.47
Dog exposure at home, *n* (%)	12 (24)	3 (33)	0.68
Cat exposure at home, *n* (%)	1 (2)	2 (22)	0.06
Allergic airway manifestations:			
Rhinitis, any reported *n* (%)	48 (96)	9 (100)	1.00
Positive NPT, *n* (%)	23 (46)	1 (11)	0.07
Rhinitis, triggered by dog, *n* (%)	35 (70)	5 (56)	0.45
Asthma, triggered by dog, *n* (%)	30 (60)	0	n.a
Asthma control test, mean (SD)	20.6 (3.37)	25.3 (1.58)	**<0.001**
Lung function and eosinophils:			
FEV1% of predicted, mean (SD)	100 (11.7)	105 (7.07)	0.18
FeNO (ppb), median (IQR)	33.5 (20–70)	29 (16–59)	0.40
BHR; PD20 < 8, umol, *n* (%)	31 (69)^†^	6 (67)	1.00
BHR; PD20 < 2, umol, *n* (%)	24 (53)^†^	1 (11)	**0.03**
Eosinophils (10^9^ × L^−1^), mean (SD)	0.44 (0.28)	0.44 (0.44)	0.96
Asthma medications:			
SABA, *n* (%)	45 (90)	1 (11.1)	**<0.001**
LABA, *n* (%)	26 (52)	0	**0.003**
Inhaled steroids, *n* (%)	34 (68)	0	**<0.001**
Inhaled steroids (μg), median (IQR)	200 (0–400)	0	**<0.001**

*Note*: Boldface indicate that p < 0.05.

*Abbreviations*: ACT, Asthma Control Test; BHR, Bronchial hyper‐reactivity; FeNO, Fraction of exhaled Nitric Oxide; FEV1, Forced expiratory volume in one second; IQR, Inter‐quartile range; LABA, Long acting beta‐2 agonist; NPT, Nasal provocation test; PD20, dose methacholine (umol) leading to a 20% drop in FEV1; Ppb, Parts per billion; SABA, Short acting beta‐2 agonist; SD, Standard deviation.

^†^ *n* = 45, Three individuals did not go through metacholine challenge because of baseline FEV1 ≤ 75%. Two did not complete the examination due to technical issues.

### 3.2 Allergic sensitization to furry animal allergen molecules in dog dander sensitized children with and without asthma

**FIGURE 1:**
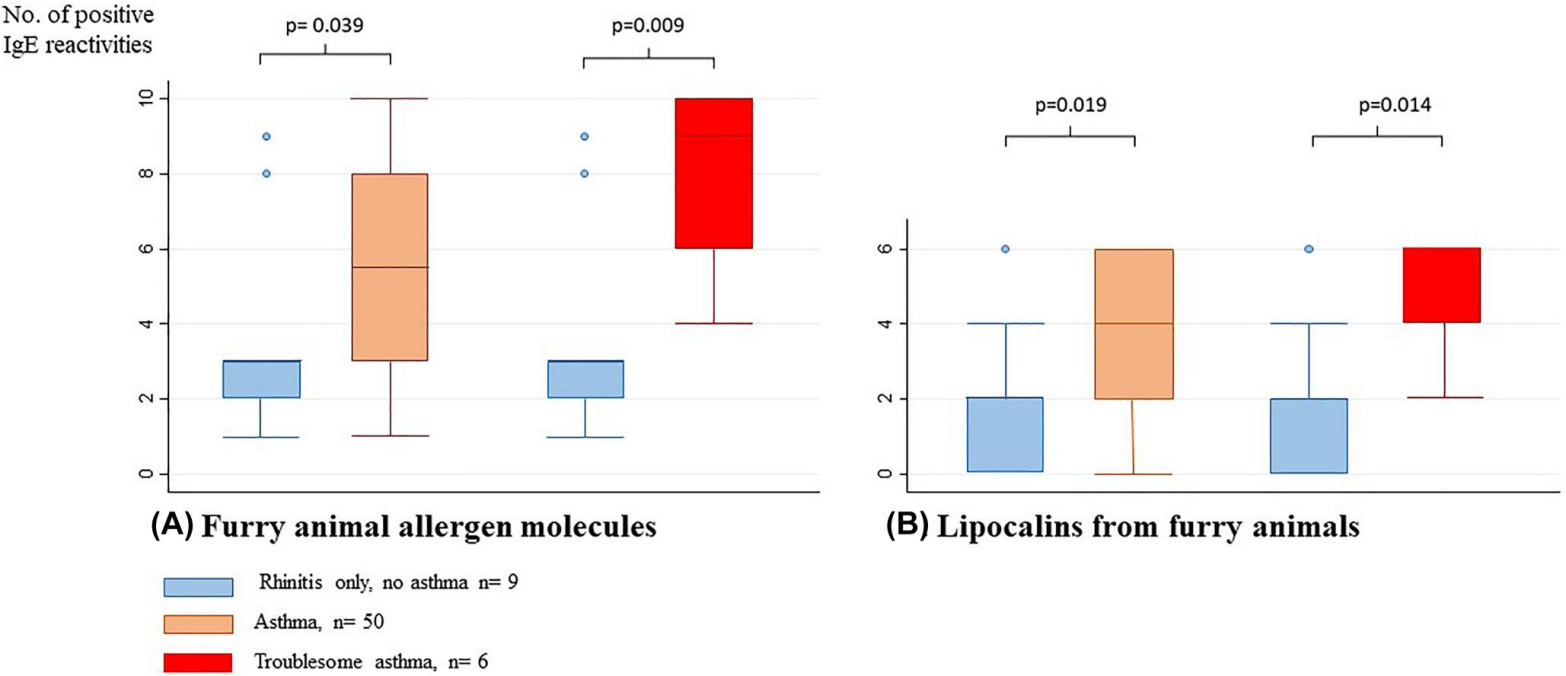
Median numbers of positive IgE reactivities (*y*‐axis) in children with no asthma (blue), asthma (orange) and troublesome asthma (red) to: A. Furry animal allergen molecules (*n* = 1–10). B. Lipocalins (*n* = 0–6). Boxes indicate interquartile ranges and whiskers maximum/minimum number of positive IgE reactivities

Children with asthma were to a larger extent polysensitized to furry animal allergen molecules and to lipocalins than children with rhinitis only (Figure 1A,B). The median number of positive IgE results to furry animal allergen molecules among children without asthma was 3, among all children with asthma 5.5 and among the children with troublesome asthma 9 (asthma vs. no asthma; *p* = 0.039; troublesome asthma vs. no asthma; *p* = 0.009) (Figure 1A). The corresponding number of positive IgE results to the lipocalins among children without asthma was 2, among all children with asthma 4 and among children with troublesome asthma 6 (Asthma vs. no asthma; *p* = 0.019; Troublesome asthma vs. no asthma; *p* = 0.014) (Figure 1B). The odds ratio for having asthma if sensitized to any lipocalin was 7.2 (95% CI: 1.44–35.9).

**FIGURE 2:**
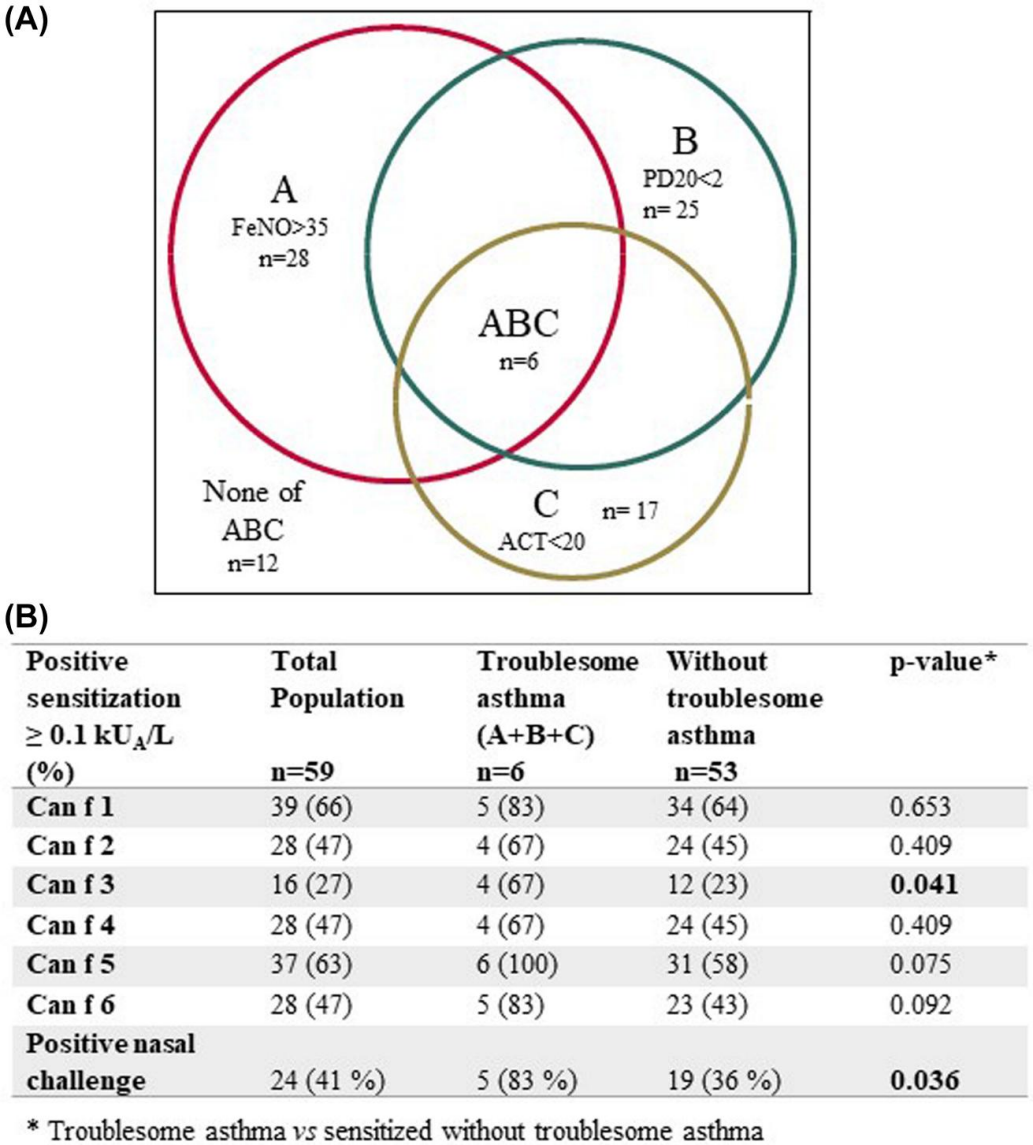
A Venn diagram showing overlapping markers for asthma severity. A, Red circle corresponding to children with fraction of exhaled nitric oxide (FeNO) > 35 ppb. B, Green circle corresponding to children with methacholine PD20 < 2 μmol. C, Yellow circle corresponding to children with Asthma Control Test (ACT) score < 20 points. B, Numbers (percentages) of positive IgE sensitizations (≥0.1 kUA/L) to dog allergen molecules and numbers (percentages) of children with positive result at nasal challenge with dog dander extract (bottom line) in children with troublesome asthma in comparison with the rest of the dog sensitized study population. Boldface indicate *p* < 0.05

There was a considerable overlap in sensitization to the cross‐reacting lipocalins Can f 6, Fel d 4 and Equ c 1 (Figure E 1, Supporting Information). Sensitization to Can f 6 was more common among dog dander sensitized children with asthma than without asthma (54% vs. 11%, *p* = 0.03). Furthermore, children with asthma showed a non‐significant trend towards being more frequently sensitized to the horse lipocalin Equ c 1 (70% vs. 33%, *p* = 0.06) and the cat lipocalin Fel d 4 (72% vs. 33%, *p* = 0.05). Besides, the IgE levels to Equ c 1 were higher among children with asthma compared to children without asthma (median 7.0 vs. 0.17 kU_A_/L, *p* = 0.01), Table 2. Following Bonferroni correction, the association between lipocalin sensitization and asthma as well as troublesome asthma (Figure 1B) and between sensitization to furry animal allergen molecules and troublesome asthma (Figure 1A) remained statistically significant, whereas the associations between single allergen molecules and asthma were no longer significant.

**TABLE 2:** Sensitization rates and IgE levels to furry animal allergen extracts and molecules among dog dander sensitized children with and without asthma

	Asthma (*n* = 50)		No asthma (*n* = 9)		*p*‐Value sensitization rates	*p*‐Value IgE levels
Allergen	Sensitization rate *n* (%)	Median level kU_A_/L (IQR)	Sensitization rate *n* (%)	Median level kU_A_/L (IQR)		
Dog dander	50 (100)	14 (3.8–47)	9 (100)	3.3 (1.3–22)	n.a.	0.08
Can f 1	35 (70)	5.9 (2.1–28)	4 (44)	7.1 (0.27–54)	0.25	0.52
Canf 2	26 (52)	6.1 (0.98–24)	2 (22)	14.6 (0.13–29)	0.15	0.59
Can f 3	15 (30)	1.8 (0.32–6.1)	1 (11)	0.21	0.42	n.a
Can f 4	25 (50)	1.1 (0.56–4.7)	3 (33)	0.58 (0.11–3.3)	0.48	0.36
Can f 5	31 (62)	5.4 (0.87–15)	6 (67)	1.1 (0.25–2.3)	1.00	0.08
Can f 6	27 (54)	0.69 (0.37–1.0)	1 (11)	0.18	**0.03**	n.a
Cat dander	49 (98)	7.7 (2.8–22)	8 (89)	25 (0.43–76)	0.28	0.64
Fel d 1	41 (82)	9.8 (4.5–27)	7 (78)	51 (0.57–100)	0.67	0.87
Fel d 2	13 (26)	0.33 (0.25–1.3)	2 (22)	18 (0.23–35)	1.00	0.43
Fel d 4	36 (72)	1.8 (0.54–7.6)	3 (33)	0.21 (0.19–100)	**0.05**	0.74
Horse dander	42 (84)	7.5 (2.1–36)	5 (56)	2.7 (0.22–17)	0.07	0.21
Equ c 1	35 (70)	7.0 (2.4–20)	3 (33)	0.17 (0.14–5.9)	0.06	**0.014**
> 3 allergens	35 (70)		2 (22)		**0.01**	
Any lipocalin	45 (90)		5 (56)		**0.02**	
Any albumin	18 (36)		2 (22)		0.70	
Total IgE		455 (360–1640)		503 (250–789)		0.43

### 3.3 Troublesome asthma and sensitization to dog, cat and horse allergen molecules

**FIGURE 3:**
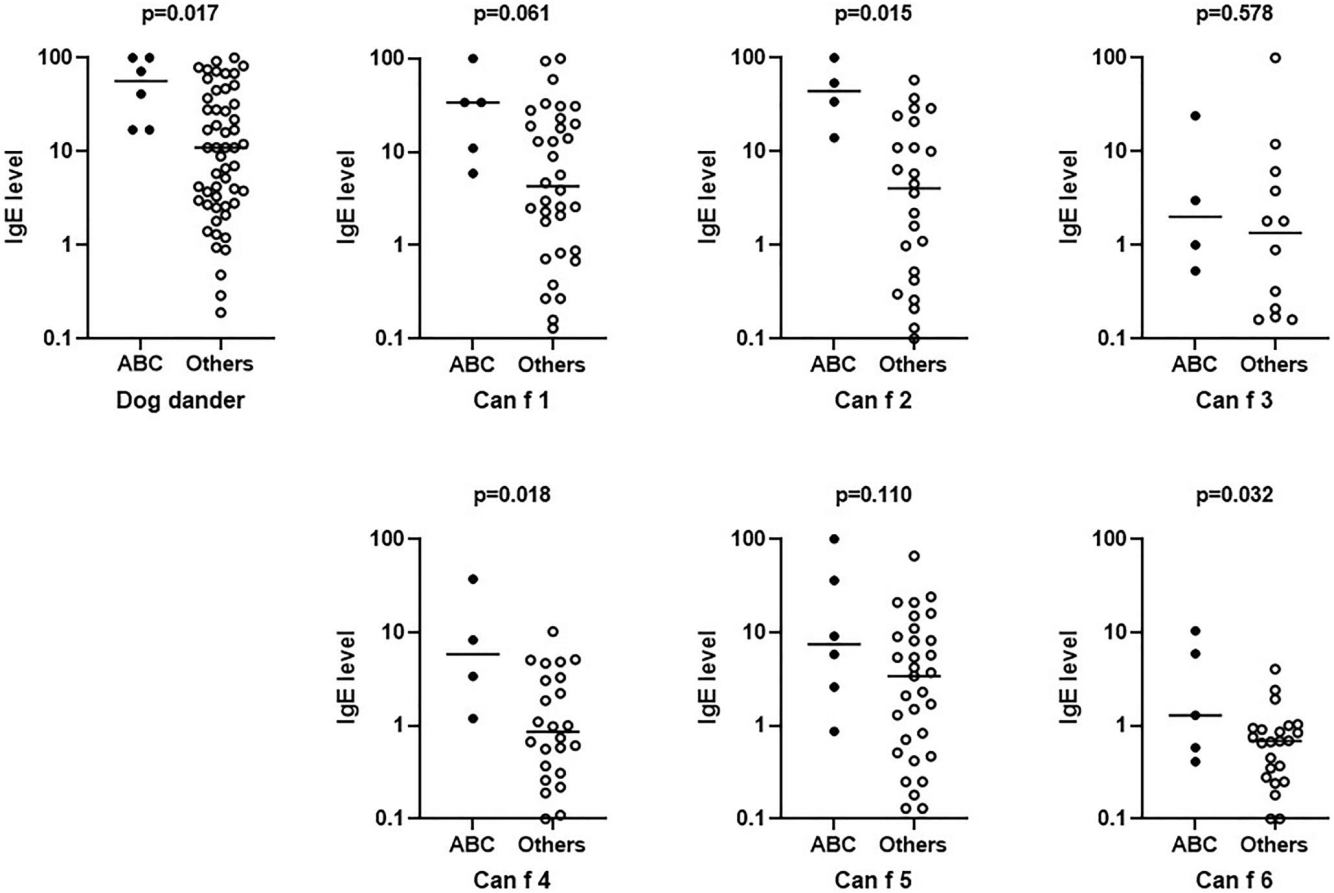
IgE levels among sensitized children with troublesome asthma “ABC” (*n* = 6), with pronounced bronchial hyperreactivity (PD20 < 2 μmol), poor asthma control (Asthma Control Test (ACT) < 20) and high levels of exhaled fraction of exhaled nitric oxide (FeNO) (>35 ppb) compared with IgE levels among sensitized children with controlled asthma or rhinitis only, “others” (*n* = 51). Horizontal bars indicate median levels. Levels above 100 kU_A_/L were set at 100 kU_A_/L

Figure 2A shows overlapping manifestations of asthma severity including asthma control, airway inflammation and bronchial hyperreactivity. Children with troublesome asthma (*n* = 6) had higher IgE levels to dog dander (57 vs. 11 kU_A_/L, *p* = 0.017) and to the dog lipocalins Can f 2 (44 vs. 4.1 kU_A_/L, *p* = 0.015), Can f 4 (5.8 vs. 0.9 kU_A_/L, *p* = 0.018) and Can f 6 (1.3 vs. 0.7 kU_A_/L, *p* = 0.03) compared to the rest of the study population (*n* = 53), see Figure 3. Sensitization to Can f 3 was more common among children with troublesome asthma (67% vs. 23%, *p* = 0.041), but their IgE levels to Can f 3 did not differ compared to the rest of the study population. Children with troublesome asthma were also more likely to have a positive nasal provocation test with dog dander extract (83% vs. 36%, *p* = 0.036), Figure 2B. No significant differences in prevalence of sensitization or IgE levels to the investigated cat or horse allergen molecules between children with troublesome asthma and the rest of the study population were found (data not shown).

### 3.4 Asthma Control Test and sensitization to furry animals

Thirty‐five percent (17/50) of the investigated children with asthma had an ACT score below 20. Asthmatics with ACT < 20 had higher IgE levels to the dog lipocalins Can f 2 and Can f 4 compared to asthmatics with ACT ≥ 20 (Table E 1, Supporting Information).

### 3.5 Airway inflammation and sensitization to furry animals

The median FeNO level was 33.5 ppb (IQR 20–70) among whom 28 (47%) had high FeNO (>35 ppb). Children with high FeNO displayed higher IgE levels towards dog dander and the dog lipocalins Can f 1 and Can f 4 than individuals with FeNO < 35 ppb (*n* = 31). Children in the high FeNO‐group were also more frequently sensitized to the horse lipocalin Equ c 1 (82% vs., 48%, *p* = 0.01), see Table E 2, Supporting Information.

### 3.6 Bronchial hyperreactivity and sensitization to furry animals

A majority, 69% (37/54), of the investigated dog dander sensitized children had a positive bronchial methacholine challenge (PD20 < 8 μmol methacholine), while 46% (25/54) showed pronounced bronchial hyperreactivity (PD20 < 2 μmol methacholine). No significant associations between sensitization rates or IgE levels to the investigated allergens and pronounced bronchial hyperreactivity were observed (Table E 3, Supporting Information).

## 4 DISCUSSION

This well characterized cohort of dog dander sensitized children represents an important and common patient group in pediatric clinics. We found that asthma was associated with polysensitization to allergen molecules and to lipocalins from furry animals. In addition, we observed that troublesome asthma was associated with a higher degree of polysensitization, increased IgE levels to the dog lipocalins Can f 2, Can f 4 and Can 6 and with a positive nasal provocation test with dog dander. Based on our findings, we suggest that children with multiple lipocalin sensitization need thorough monitoring regarding asthma management and advice regarding dog avoidance. Thus, a detailed assessment using molecular allergy diagnostics may help clinicians to evaluate the impact of allergic sensitization on asthma morbidity and thereby improve advice regarding pet exposure and immunotherapy.^23^

The association between troublesome asthma and a positive nasal provocation test confirms the concept of the united airways; that the severity of the disease in the upper airways is also reflected in the lower airways.^24^ Allergic rhinitis among pre‐school children has been associated with airway hyperresponsiveness at the age of seven.^25^ As allergic rhinitis often precedes the onset of allergic asthma and may deteriorate asthma symptoms through impaired nasal function, children with allergic rhinitis to dog should be thoroughly treated and further evaluated regarding asthma.^26^

The association between asthma and IgE to the dog lipocalins Can f 4 and Can f 6 has not been previously investigated. However, the lipocalin protein group has been pointed out as one of two protein families associated with asthma in children,^27^ and sensitization to three or more lipocalins has previously been associated with severe asthma.^28^ As IgE reactivity to Can f 6 was associated with asthma and IgE levels to Can f 4 and Can f 6 were higher among children with troublesome asthma, we suggest that these allergens could be regarded as markers for asthma among dog dander sensitized children.

In the present study, a vast majority of the children was also sensitized to allergen molecules from cat and horse, and the most common sensitizing allergen was the major cat allergen secretoglobin, Fel d 1. The IgE levels to Fel d 1 did not differ significantly between children with and without asthma or with different asthma manifestations. It has previously been reported that sensitization to Fel d 1 alone is a poor predictor of asthma in children, unless at high titers or in combination with the cat lipocalin Fel d 4.^10^

Asthma is a multi‐factorial disease. In children, the chronic inflammation is often to a large extent caused by allergic sensitization, while the bronchial hyperreactivity may depend on a broader range of factors.^29^ This may explain why we observed elevated IgE levels to dog lipocalins in children with high exhaled FeNO levels as well as insufficient asthma control, while no such associations were seen among children with bronchial hyperreactivity.^30^

The major strength of this study is that, for the first time, all clinically available dog allergen molecules were evaluated by a quantitative measure in patients that were extensively investigated regarding asthma manifestations. We also performed nasal provocation tests to elucidate associations between dog induced rhinitis and asthma, and not only sensitization patterns and asthma. We are aware of power limitations due to small sub‐groups and risk of significant findings by chance due to multiple testing. Adjusting for multiple testing in an analysis with low sample size causes unnecessary under‐powering and increases risk for type II errors to an unacceptable level,^31^ hence Bonferroni correction for multiple testing could not confirm significant results for single allergens. However, we consistently found significant associations between IgE reactivity as well as IgE levels to lipocalins and different manifestations of asthma in the unadjusted analysis. Since our results are in accordance with previous findings regarding lipocalin sensitization, we interpret these associations as clinically relevant. Furthermore, the limited size of this study do not allow multivariate analysis to reveal independent associations between sensitization and asthma manifestations. This might be a focus for future studies.

In conclusion, asthma in dog dander sensitized children is associated with polysensitization to furry animal allergen molecules and lipocalins. Furthermore, children with troublesome asthma have higher IgE levels to the dog lipocalins Can f 2, Can f 4 and Can f 6 compared to dog dander sensitized children with controlled asthma or rhinitis only. Thus, our findings suggest that a detailed assessment using molecular allergy diagnostics may help the clinicians to evaluate the impact of allergic sensitization on asthma morbidity and thereby improve advice regarding dog exposure, future treatments, and immunotherapy.

## CONFLICT OF INTEREST

Ulrika Käck report personal fees from Thermo Fisher Scientific, outside the submitted work. Marianne van Hage report personal fees from Thermo Fisher Scientific, outside the submitted work. Anna Asarnoj report personal fees from Meda/Mylan, Orion Pharma, ALK and Thermo Fisher Scientific outside the submitted work. Jon R. Konradsen has received material from Thermo Fisher to perform the IgE analysis in this project. Hans Grönlund and Gunnar Lilja declare that they have no relevant conflicts of interest.

## AUTHOR CONTRIBUTIONS

**Ulrika Käck:** Data curation; Formal analysis; Investigation; Project administration; Writing – original draft; **Marianne van Hage:** Conceptualization; Data curation; Formal analysis; Funding acquisition; Investigation; Methodology; Project administration; Writing – original draft; **Hans Gronlund:** Conceptualization; Formal analysis; Investigation; Methodology; Writing – original draft; **Gunnar Lilja:** Conceptualization; Data curation; Formal analysis; Funding acquisition; Investigation; Methodology; Supervision; Writing – original draft; **Anna Asarnoj:** Conceptualization; Data curation; Formal analysis; Investigation; Methodology; Writing – original draft; **Jon R Konradsen:** Conceptualization; Data curation; Formal analysis; Funding acquisition; Investigation; Methodology; Project administration; Writing – original draft.

## Supporting information

Supporting Information 1
