# Supplementary material for: Allergic sensitization to lipocalins reflects asthma morbidity in dog dander sensitized children
Source: Clin Transl Allergy. 2022 May 2;12(5):e12149. doi: 10.1002/clt2.12149 (PMC9058535; doi:10.1002/clt2.12149)
Supplement: Supplementary file 1 — Supporting Information 1 [file CLT2-12-e12149-s001.docx]

Figure E 1 online


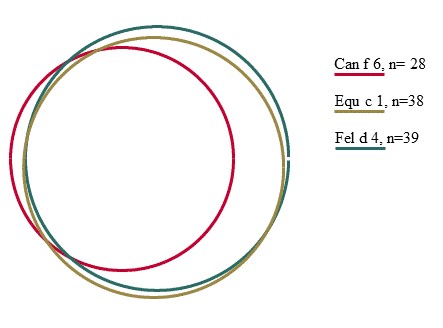


Figure E 1 online: Number of individuals with overlapping IgE reactivity to the cross-reacting lipocalins Can f 6, Equ c 1 and Fel d 4.

Table E 1, online

Sensitization rates and IgE levels among sensitized asthmatic children with poor asthma control (ACT<20) compared with sensitized asthmatic children with ACT>20.

|  | ACT<20, n=17 | | ACT>20, n=32 | |  |  |
| --- | --- | --- | --- | --- | --- | --- |
| Allergen | Sensitization rate  n (%) | IgE levels, kU_A_/L median (IQR) | Sensitization rate  n (%) | IgE levels, kU_A_/L median (IQR) | p-value  (sens. rate) | p-value  (IgE levels) |
| Dog dander | 17 (100) | 12 (5.2-68) | 32 (100) | 17 (3.4-46) | n.a | 0.55 |
| Can f 1 | 12 (71) | 12 (3.3-34) | 22 (69) | 5.2 (0.9-20) | 1.00 | 0.08 |
| Can f 2 | 8 (47) | 32 (10-56) | 18 (56) | 2.9 (0.4-11) | 0.54 | **0.005** |
| Can f 3 | 7 (41) | 0.9 (0.2-3.0) | 8 (25) | 2.8 (1.1-9.1) | 0.24 | 0.30 |
| Can f 4 | 7 (41) | 3.4 (1.2-10) | 18 (56) | 0.9 (0.4-2.2) | 0.32 | **0.03** |
| Can f 5 | 13 (76) | 5.8 (2.1-9.1) | 18 (56) | 4.8 (0.5-15) | 0.22 | 0.30 |
| Can f 6 | 9 (53) | 1.0 (0.6-1.9) | 18 (56) | 0.7 (0.4-0.9) | 0.83 | 0.09 |
|  |  |  |  |  |  |  |
| Cat dander | 17 (100) | 11 (4.4-22) | 31 (97) | 7.7 (2.4-24) | 1.00 | 0.59 |
| Fel d 1* | 14 (82) | 9.9 (4.2-26) | 26 (81) | 7.1 (4.5-32) | 1.00 | 0.53 |
| Fel d 2* | 5 (29) | 0.3 (0.2-0.3) | 8 (25) | 0.7 (0.3-5.6) | 0.75 | 0.32 |
| Fel d 4* | 14 (82) | 1.0 (0.4-9.5) | 21 (66) | 3.3 (1.2-5.6) | 0.32 | 0.20 |
|  |  |  |  |  |  |  |
| Horse dander | 15 (88) | 4.7 (2.1-36) | 26 (81) | 10 (3.6-41) | 0.70 | 0.48 |
| Equ c 1* | 12 (71) | 4.9 (2.4-27) | 22 (69) | 9.1 (3.5-16) | 1.00 | 0.72 |

*Sera that scored positive (IgE ≥0.10 kU_A_/L) for cat or horse extracts were further analyzed for IgE against allergen molecules from cat (Fel d 1, Fel d 2, Fel d 4) and horse (Equ c 1).

Table E 2, online

Sensitization rates and IgE levels among sensitized children with high FeNO (>35 ppb) vs children with normal/elevated FeNO (0-35 ppb).

|  | High FeNO(>35)  n=28 | | Normal/elevated FeN0 (<=35)  n=31 | |  |  |
| --- | --- | --- | --- | --- | --- | --- |
| Allergen | Sensitization rate  n (%) | IgE levels, kU_A_/L median (IQR) | Sensitization rate  n (%) | IgE levels, kU_A_/L median (IQR) | p-value  (sens. rate) | p-value  (IgE levels) |
|  |  |  |  |  |  |  |
| Dog dander | 28 (100) | 28 (6.45-70) | 31 (100) | 5.8 (2.5- 17) | n.a | **0.02** |
| Can f 1 | 19 (68) | 19 (4.7-34) | 20 (65) | 2.6 (0.53-11) | 0.79 | **<0.001** |
| Can f 2 | 17 (61) | 11 (2.2-29) | 11 (35) | 1.6 (0.26-11) | 0.05 | 0.16 |
| Can f 3 | 11 (39) | 1.0 (0.21-6.1) | 5 (14) | 1.8 (0.89-3.8) | 0.08 | 0.93 |
| Can f 4 | 17 (61) | 2.2 (0.98-5.1) | 11 (35) | 0.58 (0.19-1.9) | 0.05 | **0.01** |
| Can f 5 | 20 (71) | 5.4 (0.59-19) | 17 (55) | 3.4 (1.3-8.2) | 0.19 | 0.67 |
| Can f 6 | 17 (61) | 0.65 (0.37-1.0) | 11 (35) | 0.69 (0.25-1.0) | 0.05 | 0.86 |
|  |  |  |  |  |  |  |
| Cat dander | 28 (100) | 14 (2.7-61) | 29 (94) | 6.2 (2.2-12) | 0.49 | 0.12 |
| Fel d 1* | 24 (86) | 18 (3.8-74) | 24 (77) | 6.3 (3.8-19) | 0.51 | 0.26 |
| Fel d 2* | 9 (32) | 0.33 (0.23-5.4) | 6 (19) | 0.48 (0.25-0.72) | 0.26 | 0.56 |
| Fel d 4* | 22 (79) | 3.4 (1.1-11) | 17 (55) | 0.91 (0.39-4.0) | 0.05 | 0.16 |
|  |  |  |  |  |  |  |
| Horse dander | 26 (93) | 10 (3.5- 21) | 21 (68) | 3.6 (0.49-36) | **0.02** | 0.23 |
| Equ c 1* | 23 (82) | 5.1 (2.3-11) | 15 (48) | 7.6 (1.2-23) | **0.01** | 0.94 |
|  |  |  |  |  |  |  |

* Sera that scored positive (IgE ≥0.10 kU_A_/L) for cat or horse extracts were further analyzed for IgE against allergen molecules from cat (Fel d 1, Fel d 2, Fel d 4) and horse (Equ c 1).

Table E 3, online

Sensitization rates and IgE levels among children with pronounced bronchial hyperreactivity (PD20< 2 µmol) *vs* children with less bronchial hyper reactivity (PD20> 2 µmol).

|  | PD20< 2 µmol, n=25 | | PD20> 2 µmol, n=29 | |  |  |
| --- | --- | --- | --- | --- | --- | --- |
| Allergen | Sensitization rates n (%) | IgE levels, kU_A_/L median (IQR) | Sensitization rates n (%) | IgE levels, kU_A_/L median (IQR) | p-value  (Sens. rates) | p-value  (IgE levels) |
|  |  |  |  |  |  |  |
| Dog dander | 25 (100) | 17 (4.2-41) | 29 (100) | 8.9 (2.5.28) | n.a | 0.30 |
| Can f 1 | 16 (64) | 7.5 (2.2-27) | 18 (62) | 3.5 (0.9-23) | 0.88 | 0.64 |
| Can f 2 | 14 (56) | 6.1 (1.1-29) | 11 (38) | 4.5 (0.3-21) | 0.18 | 0.33 |
| Can f 3 | 9 (36) | 1.8 (0.9-3.0) | 5 (17) | 0.2 (0.2-3.8) | 0.13 | 0.29 |
| Can f 4 | 12 (48) | 2.6 (0.8-4.7) | 14 (48) | 0.7 (0.4-1.9) | 0.98 | 0.10 |
| Can f 5 | 15 (60) | 5.8 (0.5-16) | 19 (66) | 2.1 (0.7-8.2) | 0.68 | 0.35 |
| Can f 6 | 14 (56) | 0.7 (0.4-1.3) | 11 (38) | 0.7 (0.2-0.9) | 0.18 | 0.22 |
|  |  |  |  |  |  |  |
| Cat dander | 24 (96) | 10 (2.5-53) | 28 (97) | 5.9 (1.0-14) | 1.00 | 0.12 |
| Fel d 1* | 21 (84) | 12 (1.6-57) | 22 (76) | 6.0 (3.3-20) | 0.52 | 0.60 |
| Fel d 2* | 7 (28) | 0.3 (0.3-5.4) | 6 (21) | 0.5 (0.2-1.3) | 0.53 | 0.88 |
| Fel d 4* | 17 (68) | 1.8 (0.7-9.5) | 17 (59) | 1.9 (0.4-5.6) | 0.48 | 0.65 |
|  |  |  |  |  |  |  |
| Horse dander | 22 (88) | 6.4 (1.6-21) | 20 (69) | 8.4 (1.7-31) | 0.11 | 0.71 |
| Equ c 1* | 16 (64) | 6.4 (2.9-24) | 17 (59) | 5.9 (2.4-15) | 0.69 | 0.50 |

*Sera that scored positive (IgE ≥0.10 kU_A_/L) for cat or horse extracts were further analyzed for IgE against allergen molecules from cat (Fel d 1, Fel d 2, Fel d 4) and horse (Equ c 1).
